# Changes in Knowledge and Practices Regarding Tick-Borne Encephalitis Among Infectious Disease Physicians in Romania: A Repeated Cross-Sectional Survey

**DOI:** 10.3390/epidemiologia7050148

**Published:** 2026-10-21

**Authors:** Andreea Madalina Panciu, Cristina Alexandra Cheran, Traian Constantin Panciu, Gheorghita Jugulete, Adriana Hristea

**Affiliations:** 1 Faculty of Medicine, Carol Davila University of Medicine and Pharmacy, 020021 Bucharest, Romania; cristina-alexandra.constantin@drd.umfcd.ro (C.A.C.); traian-constantin.panciu@drd.umfcd.ro (T.C.P.); gheorghita.jugulete@umfcd.ro (G.J.); adriana.hristea@umfcd.ro (A.H.); 2 “Prof. Dr. Matei Bals” National Institute of Infectious Disease, 021105 Bucharest, Romania; 3 Department of Infectious Diseases, Bucharest Emergency University Hospital, 050098 Bucharest, Romania; 4 Marius Nasta Institute of Pneumology, 050159 Bucharest, Romania

**Keywords:** tick-borne encephalitis, TBEV, vaccination, physician awareness, knowledge and practices, infectious diseases, Romania

## Abstract

Background/Objectives: Tick-borne encephalitis virus (TBEV) is an emerging vector-borne pathogen in Europe, yet it may be potentially underrecognized in countries reporting few clinical cases. Despite the small number of reported TBE cases, evidence of TBEV circulation in Romania has increased in recent years. Awareness and clinical practices among infectious disease physicians remain insufficiently characterized. This study evaluated changes in knowledge and clinical practices regarding TBEV infection among infectious disease physicians in Romania between 2019 and 2025, following the introduction of TBE vaccination. Methods: We conducted a repeated cross-sectional survey using the same questionnaire in two independent cohorts of infectious disease physicians in Romania (2019, *n* = 164; 2025 *n* = 140). Knowledge of tick-borne diseases, TBEV global and local epidemiology, diagnosis, and clinical practice was evaluated and compared. Results: Recognition of TBEV cases in Romania increased significantly from 95/164 (57.9%) in 2019 to 108/140 (77.1%) in 2025 (*p* = 0.004), while awareness of vaccination as a preventive measure increased from 37.8% to 65.7% (*p* < 0.001). However, increased awareness did not translate into increased diagnostic testing: TBEV testing remained limited (25.6% in 2019 vs. 20.0% in 2025) and lack of access to serological testing was the most frequently reported diagnostic barrier (65.5% in 2019 vs. 82.1% in 2025). Awareness of alimentary transmission through unpasteurized dairy products also remained limited. Conclusions: Romanian infectious disease physicians’ awareness regarding TBEV improved between 2019 and 2025, but diagnostic practice did not improve accordingly. The persistent gap between awareness and testing capacity may contribute to under recognition of TBE and highlights the need to strengthen diagnostic access alongside physician education and vaccination strategies.

## 1. Introduction

Tick-borne encephalitis (TBE) is an emerging vector-borne infectious disease, with a growing number of cases reported in many regions of Europe and Asia, partly driven by climate-related changes [1,2]. The disease is caused by the tick-borne encephalitis virus (TBEV), a flavivirus transmitted primarily through the bite of infected *Ixodes ricinus* ticks in Europe and, less frequently, through the consumption of unpasteurized dairy products [3].

Although several European countries report substantial numbers of TBE cases annually, the epidemiological situation of TBE in Romania remains insufficiently characterized. *Ixodes ricinus*, the main TBEV vector in Europe, is widely distributed throughout the country and was identified in 97.7% of 188 sampling locations in a nationwide survey including 13,771 ticks collected from all 41 Romanian counties [4]. Evidence of TBEV circulation has been obtained from entomological investigations. TBEV isolates were identified in ticks collected from farm animals in five Romanian counties, while subsequent molecular investigations detected TBEV RNA in *Ixodes ricinus* ticks collected from vegetation and animals in Sibiu, Tulcea, and Giurgiu counties [5,6]. Serological evidence in animals further supports local viral circulation. In a study including 519 sheep from 50 localities in five north-western Romanian counties, TBEV-neutralizing antibodies were detected in 15.2% of animals, with county-level seroprevalence ranging from 2.0% to 27.7% [7]. Foodborne transmission has also been documented in Romania. In 1999 an outbreak involving at least 38 human TBE cases was recorded, with raw goat milk and goat milk products considered the probable source of infection [8].

In contrast to the evidence obtained from vectors and animals, contemporary data on human TBEV infection in Romania remain limited. Historical serological investigations suggested heterogeneous exposure, particularly in areas considered natural foci and among occupationally exposed populations, but differences in study populations and diagnostic methods limit direct comparison with more recent data [9,10]. More recently, a study of 1200 healthy blood donors from six counties in north-western Romania found only one TBEV-seropositive individual after confirmation by serum neutralization testing, corresponding to an overall seroprevalence of 0.08% (1/1200) [11]. A subsequent serosurvey including 1116 human serum samples collected from 15 localities in 10 Romanian counties found an overall TBEV seroprevalence of 0.62% (7/1116). All seropositive samples originated from a single locality in Sibiu County, where the local seroprevalence reached 9.7%, supporting the focal nature of human exposure [12].

Despite these findings, very few human TBE cases are officially reported. According to data reported to the European Centre for Disease Prevention and Control (ECDC), only one case was reported in Romania between 2019 and 2022 [13].

Studies from other European countries, including Germany and Serbia, have shown that awareness of TBE varies with local endemicity and that gaps regarding its epidemiology, transmission and prevention persist even among healthcare workers [14,15,16]. Comparable data among physicians in Romania are lacking.

In 2020, vaccination against TBE became commercially available in Romania [17], providing an additional preventive option. No national TBE vaccination program was implemented. Against this background of documented TBEV circulation, limited human epidemiological data may not fully reflect the occurrence of infection. In this context the recognition of TBE as a possible diagnosis, knowledge of its modes of transmission and geographical distribution, awareness of available diagnostic testing and familiarity with preventive measures are relevant components of clinical preparedness.

We therefore conducted repeated cross-sectional surveys among infectious disease physicians in Romania in 2019 and 2025, using the same questionnaire, to assess changes in knowledge regarding TBEV epidemiology and transmission, diagnostic and surveillance practices and awareness and use of vaccination. The 2019 survey provides a pre-availability reference point, whereas the 2025 survey reflects the situation several years after TBE vaccines became available in Romania.

## 2. Materials and Methods

### 2.1. Study Design and Setting

To assess changes over time, we conducted repeated cross-sectional surveys among infectious disease physicians and residents in Romania, administering the same questionnaire to two independent samples in 2019 and 2025.

The surveys were designed to assess knowledge and clinical practices related to TBE, including recognition of TBEV epidemiology and transmission, diagnostic testing, surveillance, and vaccination. Accordingly, the repeated cross-sectional design does not permit assessment of within-participant changes over time or causal attribution of differences between survey periods.

### 2.2. Participants and Recruitment

Eligible participants were physicians working in the field of infectious diseases in multiple counties across Romania. Physicians were recruited through professional communication channels and through direct distribution of the questionnaire during medical congresses and professional meetings. Recruitment was therefore not restricted to congress attendees or to major university centers. Respondents represented 17 counties in the 2019 cohort and 20 counties in the 2025 one. Participation was voluntary and anonymous. The questionnaire was administered electronically using Google Forms (Google LLC, Mountain View, CA, USA) and, where applicable, in paper format during professional meetings. Participants who completed the questionnaire during the respective survey periods were included in the analysis. No personally identifying information was collected. Consequently, individual participation could not be linked between the 2019 and 2025 surveys, and the analyses were conducted as comparisons of two independent cross-sectional samples rather than as a longitudinal follow-up of the same physicians.

### 2.3. Questionnaire

The same structured questionnaire was used in both survey rounds. It assessed participants’ knowledge of TBEV geographical distribution, modes of transmission, clinical manifestations, prevention and vaccination, as well as their awareness of TBE occurrence in Romania, diagnostic testing, surveillance, and barriers to laboratory diagnosis.

The questionnaire items and response options are provided in the Supplementary Material both in the original Romanian version and an English translation. For knowledge questions, responses were categorized according to the predefined correct response(s), and the proportion of participants selecting the correct response was compared between the two survey rounds.

### 2.4. Data Collection

In 2020, vaccination against TBE became commercially available in Romania [17], providing an additional preventive option, although no national TBE vaccination program was implemented. Vaccine availability introduced a new TBE-specific preventive measure into clinical practice, making physicians’ awareness of TBE and its prevention particularly relevant to assess. The 2019 survey therefore provides a pre-availability reference point, whereas the 2025 survey reflects the situation several years after the TBE vaccine became commercially available in Romania.

The two surveys used the same questionnaire and recruitment approach as far as practicable. Because the surveys were anonymous and no identifying information was collected, it was not possible to determine whether an individual respondent had participated in both survey rounds. Therefore, the study compares independent cross-sectional samples at the population level rather than measuring individual-level change.

### 2.5. Statistical Analysis

Continuous variables were summarized using medians and interquartile ranges (IQRs), while categorical variables were summarized using frequencies and percentages. Categorical variables were compared between the 2019 and 2025 cohorts using the chi-square test or Fisher’s exact test, as appropriate. A two-sided *p* value < 0.05 was considered statistically significant. Statistical analyses were performed using IBM SPSS Statistics for Windows version 25.0 (IBM Corp., Armonk, NY, USA).

### 2.6. Ethical Considerations

Information regarding the purpose of the study, voluntary participation, anonymity, use of the collected data for scientific purposes, and the right to discontinue participation was provided at the beginning of the questionnaire. Informed consent was indicated by voluntary completion and submission of the questionnaire. The study was conducted in accordance with the Declaration of Helsinki and was approved by the Ethics Committee of the National Institute for Infectious Diseases “Prof. Dr. Matei Balș” (C02279/05 March 2019; C06727/28 June2024).

## 3. Results

A total of 304 infectious disease physicians participated, 164 (53.9%) in the 2019 cohort and 140 (46.1%) in the 2025 cohort. The proportion of specialists and residents was comparable between the two cohorts, with specialists accounting for 51.8% (85/164) and 51.4% (72/140), respectively, and residents accounting for 48.2% (79/164) and 48.6% (68/140), respectively. Among specialists with available data (82/85 in 2019 and 68/72 in 2025), years of professional experience were lower in the 2025 cohort than in the 2019 cohort (median 3.5 years [IQR: 2.0–9.3], *n* = 68 vs. 9.5 years [IQR: 4.3–19.8], *n* = 82; *p* < 0.001). Chronological age was not collected.

Recognition of Lyme disease and TBEV infection as infectious diseases across Europe was high in both cohorts. Awareness of the widespread geographic distribution of Borrelia species in Europe was also high (>90%). Significant increases in awareness were observed regarding babesiosis and anaplasmosis (*p* < 0.001). Knowledge regarding the geographic distribution of TBEV improved over time, with more respondents correctly identifying Eastern and Central Europe as endemic regions. However, recognition of Russia as an endemic area declined, whereas awareness of Scandinavian countries increased.

Knowledge of the transmission routes of TBEV showed substantial improvement between surveys. Most respondents correctly identified *Ixodes ricinus* as the primary vector responsible for TBEV transmission in Europe, with the highest tick activity between April and November. Nevertheless, awareness of non-vector transmission routes remained limited. Approximately one-third of respondents correctly identified that consumption of unpasteurized dairy products from infected animals can result in TBEV infection. Knowledge of the potential burden of TBEV infection remained constant, with a quarter of respondents being unaware of the frequency of neurological involvement and associated sequelae. Regarding risk factors, respondents most frequently identified occupations involving exposure to forests, workers, and farmers as being associated with increased TBEV risk in both cohorts. Recognition of veterinarians as an at-risk group also increased over time.

Awareness of preventive measures improved significantly between 2019 and 2025. Recognition of vaccination as a preventive strategy increased from 37.8% to 65.7% (*p* < 0.001), while awareness of general protective measures, including wearing protective clothing and avoiding high-risk environments, remained high in both cohorts. Recognition of possible Borrelia burgdorferi–TBEV co-infection increased from 78.0% to 97.1% (*p* < 0.001).

Awareness of the epidemiological situation in Romania increased markedly during the study period (*p* = 0.004). The proportion of respondents who recognized the presence of TBEV infection cases in Romania increased from 57.9% to 77.1%, while the proportion reporting uncertainty regarding this matter decreased significantly. However, testing for TBEV infection remained limited, with 25.6% of respondents reporting testing in 2019 compared with 20.0% in 2025. Similarly, awareness of the existence of TBEV surveillance in Romania improved from 28.7% to 40.7% (*p* = 0.02). Limited access to serological testing was the most frequently cited barrier in both cohorts, reported by 65.5% of participants in 2019 and 82.1% in 2025, but between one-quarter and one-third considered TBEV infection rare (32.8% vs. 23.2%) or the etiological diagnosis not relevant for treatment.

Two additional questions regarding vaccination and climate change were included only in the 2025 survey. When asked whether they had recommended at one point TBE vaccination in their clinical practice, 58 respondents (42.0%) reported that they had recommended TBE vaccination in their clinical practice. Regarding the potential impact of climate change on TBEV epidemiology, most respondents (95.7%) believed that climate change could contribute to an increase in TBEV cases in Romania.

Respondents suggested in an open-ended question that awareness regarding TBEV infection could be improved primarily through educational activities for healthcare professionals including courses, conferences, and scientific presentations. Approximately 57% of respondents mentioned medical education initiatives. Public information campaigns through mass media were also frequently suggested by 33.6%, while social media and online communication were mentioned by 14.3% of participants. Additional suggestions included involvement of public health authorities and family physicians, improved access to diagnostic testing, and promotion of vaccination. Detailed results are presented in Table 1.

## 4. Discussion

The present study demonstrates a substantial improvement in knowledge and awareness regarding tick-borne encephalitis virus (TBEV) infection among Romanian infectious disease physicians between 2019 and 2025. Despite the progress, important knowledge gaps persist, particularly regarding transmission routes, local epidemiology, and diagnostic practices. The composition of participants was comparable between the two survey waves, with a balanced proportion of specialists and residents in both survey waves.

Awareness regarding Lyme disease and TBEV infection as important tick-borne diseases present in Europe remained consistently high in both survey waves. The higher level of awareness regarding Lyme disease compared with TBEV observed in our study mirrors findings from other European surveys, where Lyme borreliosis is generally perceived as a more familiar and clinically relevant tick-borne infection. In a large household survey of more than 50,000 individuals from 20 European countries, conducted among a representative sample of the general population in 2020, awareness of Lyme disease exceeded awareness of TBE in most regions, particularly in countries where TBE is not considered highly endemic. Also, the TBEV awareness level in the general population was on average 74% in endemic countries and only 30% in non-endemic countries [14].

TBEV is widely distributed across Europe and northern Asia, with most reported cases occurring in Central and Eastern Europe and in Russia, while endemic foci are also well established in the Scandinavian countries [13,18]. In our survey, however, recognition of these endemic regions remained incomplete, particularly regarding Scandinavia. These findings suggest that knowledge regarding the geographical distribution of TBEV among infectious disease physicians remains incomplete and may have implications for travel medicine counselling and vaccination recommendations for individuals travelling to endemic regions.

Particular attention should be given to foodborne TBEV transmission, as fewer than one-third of respondents correctly identified consumption of unpasteurized dairy products as a potential route of infection in either survey wave. This finding is important because foodborne outbreaks of TBE have been documented in several European countries, including Romania, and represent a well-established, although less frequently discussed, mechanism of infection [8,21]. This knowledge gap should be considered a priority topic in future continuing medical education, particularly when counselling patients with occupational, dietary, or rural exposure histories. Educational interventions should address not only tick-bite prevention but also recognition of non-vector transmission routes and their relevance to the Romanian epidemiological context.

Although our survey included only infectious disease physicians, comparable knowledge gaps have also been reported in neighboring Serbia. In a questionnaire-based study including 642 participants from the general population, professionally tick-exposed individuals and healthcare workers demonstrated significantly higher knowledge scores compared with the general population (*p* < 0.001). However, important deficiencies regarding TBE epidemiology, transmission routes, vaccination and preventive measures remained across all participant groups, while preventive practices also differed significantly between groups (F = 12.920, *p* < 0.001) [15]. Likewise, our study identified persistent gaps regarding the geographical distribution of TBEV, alimentary transmission and access to diagnostic testing. Together, the results indicate that educational needs extend beyond the general population and remain relevant even among healthcare professionals. Similar surveys involving the Romanian general population and other categories of healthcare workers could provide valuable information for future public health interventions.

In our survey, recognition of TBEV infection in Romania increased from 57.9% in 2019 to 77.1% in 2025; nevertheless, only 20.0% of respondents in 2025 reported testing for TBEV in their institutions. Among respondents who did not test, lack of access to serological testing was the most frequently reported barrier and increased from 65.5% to 82.1%. These findings suggest that the low number of laboratory-confirmed cases cannot necessarily be interpreted as evidence of negligible TBEV circulation. The coexistence of limited diagnostic capacity and low reported incidence may create a self-reinforcing cycle in which a disease perceived as uncommon is tested for infrequently, while infrequent testing limits opportunities for laboratory confirmation and subsequent surveillance reporting. Although our survey cannot establish that physicians’ perceptions of TBE risk are directly determined by national surveillance figures, the finding that 23.2% of respondents in 2025 still considered TBEV infection rare highlights the importance of interpreting reported incidence in the context of diagnostic availability. The available Romanian serological evidence further supports the need for caution when interpreting the apparent low incidence of TBE [12]. Furthermore, a substantial proportion of acute viral meningitis cases in Romania remain without an identified aetiology [22]. Taken together, these findings indicate that strengthening access to laboratory diagnosis is essential for improving clinical recognition and surveillance of TBE in Romania.

Awareness regarding the existence of a national TBEV surveillance system in Romania increased significantly between surveys. Even so, still fewer than half of respondents were aware of the surveillance system, indicating that knowledge regarding a national reporting system remains incomplete. Similar concerns regarding diagnostic accessibility and surveillance quality have been raised across Europe, where differences in testing practices and case definitions continue to affect reported incidence rates [8,13].

Awareness regarding preventive measures also improved over time, particularly regarding repellents, vaccination, and permethrin-treated clothing. One of the most important and encouraging findings of our survey is the marked increase in recognition of vaccination as the primary preventive measure against TBEV infection. Awareness of vaccination increased from 37.8% in 2019 to 65.7% in 2025, following the introduction of TBE vaccination in Romania in 2020. Although this represents considerable progress, this proportion remains lower than expected among infectious disease specialists. Vaccination is considered the most effective preventive intervention against TBE, with excellent real-world effectiveness and strong evidence supporting its role in reducing disease incidence in endemic regions [23].

In a cross-sectional survey conducted among primary care physicians in Bavaria and Baden-Wuerttemberg, Germany, a highly endemic European region, from 2321 physicians that participated, 91% reported awareness of national vaccination recommendations, while 98% considered their knowledge regarding the risks and benefits of TBE vaccination to be adequate. In the same study 97% of physicians reported offering TBE vaccination, 67% provided vaccination counselling during the first consultation with new patients, and 64% actively reminded patients when booster doses were due [16]. By comparison, these findings contrast with our results, where although recognition of vaccination as a preventive measure increased substantially, only 42% of respondents reported having ever recommended TBE vaccination in clinical practice. This difference likely reflects the longer history of TBE endemicity, higher reported disease burden, and more established vaccination recommendations in southern Germany compared with Romania.

The German study also highlights that even in regions with high physician awareness and routine vaccine availability, additional educational tools remain necessary. Approximately 24% of surveyed physicians expressed a need for further information or educational materials regarding tick-borne diseases and their prevention [16]. This is consistent with our findings, in which Romanian infectious disease physicians frequently suggested educational activities for healthcare professionals, public awareness campaigns, and improved access to diagnostic testing and vaccination as strategies to increase TBEV awareness. Together, these observations suggest that professional education remains relevant even in settings with established TBE vaccination programs, while in Romania such interventions may be even more important given the lower frequency of vaccine recommendation and limited access to diagnostic testing.

Although conducted in an academic population rather than among healthcare professionals, a recent pan-European knowledge, attitudes and practices survey including 9401 participants from 22 European countries also demonstrated that knowledge does not necessarily translate into preventive behavior. Despite generally good awareness regarding tick-borne diseases, the study identified important differences in preventive practices between countries and highlighted the need for continuous educational initiatives adapted to local epidemiology [24]. Similarly, in our study, although awareness regarding TBE and vaccination improved substantially over time, recommendation of TBE vaccination in their clinical practice is low, suggesting that increased knowledge alone may be insufficient to influence routine clinical behavior.

A multinational survey conducted in 20 European countries identified perceived personal risk as the strongest predictor of TBE vaccine uptake while low perceived risk represented the most frequently reported reason for remaining unvaccinated. These findings suggest that awareness of vaccination alone may not be sufficient to translate into routine vaccine recommendation if both physicians and patients perceive TBE as an uncommon disease [14]. In Romania, where only sporadic cases have been officially reported and vaccination has been available only since 2020, this low perception of disease risk may contribute to the limited implementation of vaccination in clinical practice.

Overall, our results indicate that awareness of TBEV among Romanian infectious disease physicians has improved over the past six years, probably reflecting increased scientific interest in the disease and its expanding epidemiology across Europe. However, persistent gaps regarding transmission routes, vaccination, national epidemiology, and diagnostic availability indicate the need for targeted educational initiatives and strengthened surveillance systems. Improving physician knowledge is likely to contribute not only to earlier diagnosis and more accurate reporting but also to enhanced prevention efforts through vaccine advocacy and patient education.

A major strength of this study is the direct comparison of physician knowledge across two time points using a similar questionnaire, allowing temporal changes in awareness to be assessed.

However, several limitations should be acknowledged. Recruitment was mostly conducted through professional communication channels and during medical congresses, which may have resulted in overrepresentation of physicians working in larger academic or university-affiliated medical centers and underrepresentation of clinicians practicing in smaller regional or rural hospitals. Consequently, the findings may not fully reflect diagnostic practices or perceptions among physicians working in areas where tick exposure and rural occupational activities are more common. Participation was voluntary, potentially introducing selection bias, and respondents may have had a greater interest in infectious diseases and vector-borne infections than non-respondents. The cross-sectional design does not allow causal inference regarding factors responsible for the observed improvements. In addition specialists in the 2025 cohort had fewer years of professional experience than those in the 2019 cohort, which may have contributed to some of the observed differences between the two survey periods. However, chronological age was not collected, and the present study cannot determine the extent to which differences in professional experience influenced the observed findings. Finally, self-reported knowledge may not fully reflect actual clinical practice or diagnostic behavior. In addition, because the surveys were anonymous, individual respondents could not be linked across survey rounds. Therefore, we could not determine whether some physicians participated in both 2019 and 2025. Although the study was designed as a repeated cross-sectional survey rather than a longitudinal cohort, prior participation could theoretically have influenced responses among repeat participants through familiarity with the questionnaire. The magnitude and direction of any such effect cannot be determined from the available data. Future studies using geographically stratified recruitment and broader inclusion of regional healthcare facilities would help determine whether the observed knowledge and diagnostic gaps are consistent across different healthcare settings in Romania.

## 5. Conclusions

This repeated cross-sectional survey demonstrates a substantial improvement in knowledge regarding tick-borne encephalitis among infectious disease physicians in Romania over the six-year study period. Recognition of TBEV infection cases in Romania increased from 57.9% in 2019 to 77.1% in 2025, and recognition of vaccination as a preventive measure from 37.8% to 65.7%. However, this improvement did not translate into diagnostic practice, TBEV testing remained limited (25.6% in 2019 vs. 20.0% in 2025), and lack of access to serological testing was the most frequently reported barrier (65.5% in 2019 vs. 82.1% in 2025). In 2025, nearly one-quarter of non-testing respondents (23.2%) still considered TBEV infection rare. In this context, the very low number of officially reported TBE cases in Romania should be interpreted with caution.

Important knowledge gaps also persist. Less than one-third of respondents recognized alimentary transmission through unpasteurized dairy products (25.0% in 2019 vs. 29.3% in 2025). Furthermore, although recognition of vaccination improved markedly, only 42.0% of respondents in 2025 reported having ever recommended TBE vaccination in clinical practice, highlighting that improved knowledge does not necessarily translate into changes in clinical practice.

Strengthening continuous medical education, expanding access to laboratory diagnosis, and reinforcing national surveillance and public health initiatives may improve the recognition, reporting, and prevention of TBE in Romania.

## Figures and Tables

**Table 1:** Knowledge and clinical practices regarding tick-borne encephalitis among infectious disease physicians in Romania, 2019 and 2025.

	2019 *N* = 164	2025 *N* = 140	*p*-Value
**Participant characteristics**			
Professional status, *n* (%)			
Infectious diseases specialist	85 (51.8)	72 (51.4)	—
Resident	79 (48.2)	68 (48.6)	—
Professional experience among specialists, median (IQR), years * (*N* = 82/2019; *N* = 68/2025)	9.5 (4.3–19.8)	3.5 (2.0–9.3)	<0.001
**Knowledge on**			
**Tick-borne diseases in Europe**			
Lyme disease	162 (98.8)	140 (100)	0.501
TBEV	149 (90.9)	134 (95.7)	0.096
Babesiosis	59 (36.0)	80 (57.1)	<0.01
Anaplasmosis	45 (27.4)	68 (48.6)	<0.01
TBEV infection in Romania	95 (57.9)	108 (77.1)	0.004
Geographical distribution of TBEV **			
Russia	94 (57.3)	64 (45.7)	0.044
Central Europe	105 (64.0)	98 (70.0)	0.270
Eastern Europe	108 (65.9)	100 (71.4)	0.297
Scandinavian countries	40 (24.4)	48 (34.3)	0.05
**TBEV surveillance system in Romania**	47 (28.7)	57 (40.7)	0.027
**TBEV transmission routes**			
Transmission via tick bite	160 (97.6)	138 (98.6)	0.528
Transmission via unpasteurized dairy	41 (25.0)	41 (29.3)	0.401
**TBEV vector *Ixodes ricinus***	144 (87.8)	127 (90.7)	0.416
**Tick activity seasonality**			
April–November	160 (97.6)	137 (97.7)	1
September–November	2 (1.2)	4 (2.9)	0.419
**Coinfection TBEV–*Borrelia burgdorferi***	128 (78.0)	136 (97.1)	<0.001
**Occupational risk for TBEV infection**			
Farmer	150 (91.5)	125 (89.3)	0.519
Hunter	144 (87.8)	129 (92.1)	0.213
Forester	156 (95.1)	139 (99.3)	0.033
Veterinarian	94 (57.3)	100 (71.4)	0.011
**Estimated proportion of TBEV infections resulting in neurologic disease**			
<50% of clinical cases	96 (58.5)	86 (61.4)	0.871
>50% of clinical cases	24 (14.6)	17 (12.1)	
Don’t know	42 (25.6)	37 (26.4)	
**Preventive measures for tick-bite**			
DEET repellents	95 (57.9)	109 (77.9)	<0.001
Permethrin-treated clothing	58 (35.4)	62 (44.3)	0.113
Avoid forest/high grass	141 (86.0)	127 (90.7)	0.202
Protective clothing	153 (93.3)	134 (95.7)	0.360
Vaccination as prevention	62 (37.8)	92 (65.7)	<0.001
**Testing for TBEV**	42 (25.6)	28 (20.0)	0.247
**Reason for non-testing for TBEV**			
No access to serology	78 (65.5)	92 (82.1)	
Infection is considered rare	39 (32.8)	26 (23.2)	
Diagnosis not relevant for treatment	10 (8.4)	4 (3.6)	

Values are presented as *n* (%). The denominator was always 164 and 140, * except for years in professional experience, specialists with available data (82/85 in 2019 and 68/72 in 2025). *p*-values were calculated using the chi-square test or Fisher’s exact test, as appropriate. Abbreviations: TBEV, tick-borne encephalitis virus; DEET, N,N-diethyl-meta-toluamide. ** Geographical distribution based on published sources [13,18,19,20].

## Data Availability

The original contributions presented in this study are included in the article/Supplementary material. Further inquiries can be directed to the corresponding author.

## Supplementary Materials

The following supporting information can be downloaded at: https://www.mdpi.com/article/10.3390/epidemiologia7050148/s1. File S1: Study questionnaire (Romanian and English versions).

## Abbreviations

The following abbreviations are used in this manuscript:

TBE	tick-borne encephalitis
TBEV	tick-borne encephalitis virus
ECDC	European Centre for Disease Prevention and Control
IQR	interquartile range
DEET	N,N-diethyl-meta-toluamide
