# Supplementary material for: Changes in Knowledge and Practices Regarding Tick-Borne Encephalitis Among Infectious Disease Physicians in Romania: A Repeated Cross-Sectional Survey
Source: Epidemiologia (Basel). 2026 Oct 21;7(5):148. doi: 10.3390/epidemiologia7050148 (PMC13703034; doi:10.3390/epidemiologia7050148)
Supplement: Supplementary file 1 [file epidemiologia-07-00148-s001.zip › epidemiologia-4538087-supplementary-1.pdf]

Part I – Original Romanian questionnaire

Chestionar INBI Prof Dr. "Matei Bals"

Sunteți invitat(ă) să participați la un studiu de cercetare care evaluează cunoștințele și practicile clinice privind bolile transmise prin căpușe în rândul medicilor specialiști și rezidenți de boli infecțioase din România.

Participarea este în întregime voluntară. Chestionarul este anonim și nu vor fi colectate date cu caracter personal care să permită identificarea participanților. Informațiile obținute vor fi utilizate exclusiv în scopuri științifice și de cercetare și pot fi incluse în publicații sau prezentări științifice.

Puteți alege să nu participați sau puteți întrerupe completarea chestionarului în orice moment.

Prin completarea și transmiterea voluntară a acestui chestionar, confirmați că ați citit și înțeles informațiile de mai sus și că sunteți de acord să participați la studiu.

1. Sunteti:

- Specialist Boli Infecțioase
- Rezident Boli Infectioase

2. Daca sunteți medic specialist, ce vechime aveti?

____

3. Ce boli pot fi transmise la om prin mușcătura de căpușa in Europa?

- Boala Lyme
- Encefalita transmisa de căpușă
- Febra pătată a munților stâncoși
- Babesioză
- Febra butunoasă Mediteraneană
- Febra recurentă
- Febra hemoragică Crimeea-Congo
- Tularemie
- Anaplasmoză

4. Credeți că există cazuri de Tick-Borne Encephalitis Virus (TBEV) în România:

- Da
- Nu
- Nu știu

5.Cum poate fi transmisă infecția cu TBEV (Tick-borne encephalitis virus)?:

- Prin mușcătura de căpușă

- Prin consum de lapte si alte produse lactate nepasteurizate
- Prin contact cu resturi de căpușa infectata
- Prin prelucrare in laborator a resturilor din căpușă
- Transmitere de la om la om
- Transmitere materno-fetală

6. Ce tip de căpușă transmite În România infecția cu TBEV?

- Amblyoma americanum
- Dermacentor andersoni
- Ixodes Ricinus

7. In ce perioada din an sunt mai active capusele?

- Septembrie-Noiembrie
- Decembrie – Februarie
- Aprilie– Noiembrie

8. Poate exista co-infectie Lyme – TBEV, post mușcătura de căpușa?

- Da
- Nu

9. Care este procentul de pacienți care experimentează simptome ce implică sistemul nervos central

(faza secundară a bolii)?

- < 50%
- > 50%
- Nu știu

10.Ce ocupații sunt considerate cu risc crescut pentru infectie cu TBEV?

- Fermier
- Vânător
- Pădurar
- Medic veterinar
- Măcelar
- Bucătar

11. Ce zone din lume sunt considerate cu risc crescut pentru infecția cu TBEV?

- Rusia
- Europa centrala
- Europa de Est
- Tarile Scandinave
- SUA
- Japonia

- America latina

12. In Romania exista supraveghere obligatorie pentru TBEV?

- Da
- Nu

13. Ce metode sunt eficiente pentru prevenirea infecției cu TBEV:

- Utilizarea de repelenti pe baza de DEET
- Impregnarea hainelor cu permethrin
- Evitarea zonelor împădurite și cu iarba înaltă
- Acoperirea zonelor expuse de piele utilizând haine cu maneci lungi, pantaloni lungi, palarii
- Vaccinarea

14. Testati pentru infectia cu TBE cazurile de meningită, meningita-encefalita, meningoradiculită sau

meningomielită din clinica dumneavoastra?

- Da
- Nu

15. Dacă nu, de ce?

- Infecția cu TBEV este rară
- Nu am acces la investigația serologică
- Stabilirea diagnosticului meningitei, meningita-encefalitei, meningoradiculitei sau
- meningomielitei nu este importantă din perspectiva tratamentului etiologic
- Alt motiv (detaliati)

....

16. Ati recomandat vaccinarea pentru TBEV?

- Da
- Nu

17. Credeti ca schimbarile climatice ar putea influenta cresterea cazurilor de TBEV in Romania?

- Da
- Nu

18. Ce metode credeti ca ar fi utile pentru cresterea cunostiintelor legate de TBEV in Romania?

....

19 . Judetul in care profesati..

Va multumim!

Part 2 - Part II – English translation of the original Romanian questionnaire

Participant Information and Informed Consent

You are invited to participate in a research study assessing knowledge and clinical practices regarding tick-borne diseases among infectious diseases specialists and residents in Romania.

Participation is entirely voluntary. The questionnaire is anonymous, and no personally identifiable information will be collected. The information obtained will be used exclusively for scientific and research purposes and may be included in scientific publications or presentations.

You may choose not to participate or may discontinue completion of the questionnaire at any time.

By voluntarily completing and submitting this questionnaire, you confirm that you have read and understood the information above and agree to participate in the study.

1. You are:

- Infectious Diseases Specialist
- Infectious Diseases Resident

2. If you are a specialist, how many years have you been practicing as a specialist?

---

3. Which diseases can be transmitted to humans through tick bites in Europe?

- Lyme disease
- Tick-borne encephalitis
- Rocky Mountain spotted fever
- Babesiosis
- Mediterranean spotted fever
- Relapsing fever
- Crimean-Congo hemorrhagic fever
- Tularemia
- Anaplasmosis

4. Do you think that cases of tick-borne encephalitis virus (TBEV) infection occur in Romania?

- Yes
- No
- I don't know

5. How can TBEV (tick-borne encephalitis virus) infection be transmitted?

- Through a tick bite
- Through the consumption of unpasteurized milk and other dairy products

- Through contact with material from infected ticks
- Through laboratory handling of tick material
- Human-to-human transmission
- Maternal-fetal transmission

6. Which tick species transmits TBEV infection in Romania?

- Amblyomma americanum
- Dermacentor andersoni
- Ixodes ricinus

7. During which period of the year are ticks most active?

- September–November
- December–February
- April–November

8. Can Lyme disease–TBEV co-infection occur following a tick bite?

- Yes
- No

9. What proportion of patients experience symptoms involving the central nervous system (the second phase of the disease)?

- <50%
    - 50%
- I don't know

10. Which occupations are considered to be at increased risk of TBEV infection?

- Farmer
- Hunter
- Forester
- Veterinarian
- Butcher
- Cook

11. Which regions of the world are considered to be at increased risk of TBEV infection?

- Russia
- Central Europe
- Eastern Europe
- Scandinavian countries
- USA
- Japan
- Latin America

12. Is there mandatory surveillance for TBEV in Romania?

- Yes
- No

13. Which methods are effective in preventing TBEV infection?

- Use of DEET-based repellents
- Wearing permethrin-treated clothing
- Avoiding wooded areas and areas with tall grass
- Covering exposed skin by wearing long-sleeved clothing, long trousers, and hats
- Vaccination

14. Do you test patients with meningitis, meningoencephalitis, meningoradiculitis, or meningomyelitis in your clinical practice for TBEV infection?

- Yes
- No

15. If not, why?

- TBEV infection is rare
- I do not have access to serological testing
- Establishing the diagnosis of meningitis, meningoencephalitis, meningoradiculitis, or meningomyelitis is not important from the perspective of etiological treatment
- Other reason (please specify):

………

16. Have you recommended TBEV vaccination?

- Yes
- No

17. Do you think that climate change could contribute to an increase in TBEV cases in Romania?

- Yes
- No

18. What methods do you think would be useful for increasing knowledge and awareness of TBEV in Romania?

………

19. County in which you practice:

………

Thank you!
